# Maternal Mental Health Care Matters: The Impact of Prenatal Depressive and Anxious Symptoms on Child Emotional and Behavioural Trajectories in the French EDEN Cohort

**DOI:** 10.3390/jcm12031120

**Published:** 2023-01-31

**Authors:** Kadri-Ann Kallas, Ketevan Marr, Simi Moirangthem, Barbara Heude, Muriel Koehl, Judith van der Waerden, Naomi Downes

**Affiliations:** 1 Social Epidemiology Research Team, Institut Pierre Louis d’Épidémiologie et de Santé Publique, INSERM U1136, Sorbonne Université, 75012 Paris, France; 2 Orchad Team, Epidemiology and Biostatistics Sorbonne Paris Cité Center, INSERM UMR1153, INRAE, Université de Paris, 75001 Paris, France; 3 Neurogenesis and Pathophysiology Group, Neurocentre Magendie, INSERM U1215, Université de Bordeaux, 33000 Bordeaux, France

**Keywords:** socioemotional and behavioural problems, trajectories, child development, prenatal stress, anxiety, depression, mental health care, pregnancy

## Abstract

Few studies have investigated longitudinal trajectories of child socioemotional and behavioural development in relation to maternal prenatal mental health exposure or taken into consideration of the potential buffering effects of psychological intervention during pregnancy. Using data from 1135 mother–child dyads from the EDEN cohort from the general French population, Group-based trajectory modelling was used to model trajectories of behavioural and emotional characteristics measured at four timepoints via a parent-administered Strengths and Difficulties Questionnaire. Using propensity scores and inverse probability weighting to account for confounding factors, multinomial logistic regressions were used to quantify the associations with maternal symptoms of prenatal depression and anxiety. Stratified analyses were conducted by reporting psychologist and psychiatrist consultations during pregnancy. Compared to those without psychological problems, children of mothers with comorbid anxiety and depression retained a higher probability of following high and intermediate trajectories of emotional problems and a high trajectory of conduct problems throughout childhood. This increased risk was not present in the children of mothers who sought support through a prenatal psychologist or psychiatrist consultation. This article adds to a body of evidence underlining the importance of mental health care for expecting mothers.

## 1. Introduction

During pregnancy, between 10% and 25% of all women in high-income countries [1,2] and up to 65% in low- and middle-income countries [3] experience mental health problems, including symptoms of depression and anxiety. Unaddressed, these experiences can have serious consequences for expectant mothers, who have been shown to have poor self-care, engage in less prenatal care, have less gestational weight gain, are more likely to smoke or use alcohol, and have greater rates of self-injurious behaviour, which is one of the leading causes of maternal mortality [4,5]. Prenatal mood disorders are also associated with numerous obstetric risks (e.g., preterm birth, low birth weight, and intrauterine growth restriction) and short-term neonatal effects, such as increased distress after delivery and disrupted sleep patterns [6,7].

Aside from the detrimental consequences for the mother herself, experiences of prenatal mental health problems have been associated with maladaptive child outcomes through alterations in both the intra-uterine and postnatal environment [8,9]. Without intervention, poor maternal prenatal mental health problems can persist throughout pregnancy and the postpartum, thus increasing the risk of offspring’s future developmental and behavioural problems [10,11]. A variety of evidence from clinical, pre-clinical, and neuroimaging studies links prenatal anxiety and depression exposure to numerous adverse child outcomes such as difficult temperament [12], behavioural dysregulation, internalising and externalising problems [13]. While these outcomes are often temporary in nature, there are indications that they may also persist throughout early childhood and even in adulthood [10,11,14]. For example, recent evidence has demonstrated associations between prenatal maternal mental health problems and long-term emotional and behavioural difficulties in offspring [15,16,17]. Overall, maternal prenatal anxious and depressive symptomatology consistently predict higher levels of behavioural and emotional symptoms until adolescence [16,18]. This continuous effect of prenatal exposure on later child emotional and behavioural difficulties appears to remain significant even after controlling for postnatal stressors, highlighting the unique effect of the prenatal environment on adverse child outcomes [9,15,16].

Given that this association has been well documented, it is important to identify measures that can be implemented preventively in a clinical setting to protect against or reverse these unfavourable developmental trajectories. One potential factor that might impact variations in child outcomes over time may be whether the mother receives mental health care during pregnancy. Most international guidelines agree that psychotherapy, especially cognitive behavioural therapy (CBT) and interpersonal therapy, should be considered as first-line interventions for mild to moderate depression and anxiety, both during pregnancy and the postpartum period [19]. The use of psychotherapy avoids in utero medication exposure for the foetus, has a very low risk of adverse effects, and seems to be preferred by pregnant women over pharmacological interventions [20].

However, it is not yet clear whether these interventions are also able to positively impact child developmental outcomes over time. There is a paucity of research exploring the contribution of perinatal depression and anxiety interventions in reducing adverse child outcomes [21], with current studies mostly focusing on outcomes related to postpartum depression. Indeed, reviews show mixed support for the efficacy of maternal depression care on child outcomes [22,23]. Some smaller studies have indicated that prepartum interventions for depression and subsequent improvement in a mother’s mental health status are associated with improved infant outcomes [24,25] and a randomised controlled trial [26] found neurobiological differences in children of mothers who received CBT in pregnancy in areas involved in cognitive function and stress response, though no behavioural differences were observed. This reinforces the importance of measuring child outcomes rather than solely relying on the improvement of maternal symptoms or the mother–child relationship to detangle this association [27].

Overall, few studies have investigated longitudinal trajectories of child socioemotional and behavioural development in relation to exposure to maternal prenatal mental health or taken into consideration the potential buffering effects of psychological support during pregnancy in this association [28,29]. To our knowledge, this present study is the first to investigate whether exposure to maternal prenatal depression or anxiety symptoms is associated with allocation to longitudinal trajectories of child emotional and behavioural problems between ages 3 and 11 in a sample from the general population. Additionally, the paper explores whether a possible association differs for children whose mothers accessed mental health support by visiting a psychologist or psychiatrist during pregnancy. We hypothesise that there will be a positive association between mothers’ prenatal depressive and anxiety symptomatology and offspring’s odds of allocation to different unfavourable emotional and behavioural longitudinal trajectories throughout childhood and that the magnitude of this association will be reduced by timely mental health care of psychological symptoms during pregnancy.

## 2. Materials and Methods

### 2.1. The EDEN Cohort

This study uses data from the EDEN cohort, which consists of mother–child pairs recruited antenatally before 24 weeks amenorrhea in 2003–2006 from 2 university hospitals in France (Nancy and Poitiers) during prenatal visits to the departments of Obstetrics and Gynaecology. On average, women were enrolled at 15 weeks of amenorrhea (range: 8–26). Exclusion criteria included multiple pregnancies, pre-pregnancy diabetes, intention to deliver outside the university hospitals or move out of the study region within the next 3 years, and the inability to speak and read French. Among 3758 eligible women invited to participate in the study, 53% (*n* = 2002) were enrolled. Compared to a nationally representative sample of pregnant women in France in 2003, EDEN study participants had higher educational attainment but were similar in other key sociodemographic characteristics and birth outcomes. Birth data were obtained from 1899 mother–infant pairs. During pregnancy and after birth, sociodemographic and biomedical data on the mother and child were gathered from medical records, face-to-face interviews with the mother, and mother’s self-completed questionnaires. The longitudinal EDEN cohort received approval from the ethics committee of Kremlin Bicêtre and from CNIL (Commission Nationale Informatique et Liberté), the French data privacy institution. Written consent was obtained from the mothers for themselves at inclusion and for the children after delivery [30].

**Figure 1:**
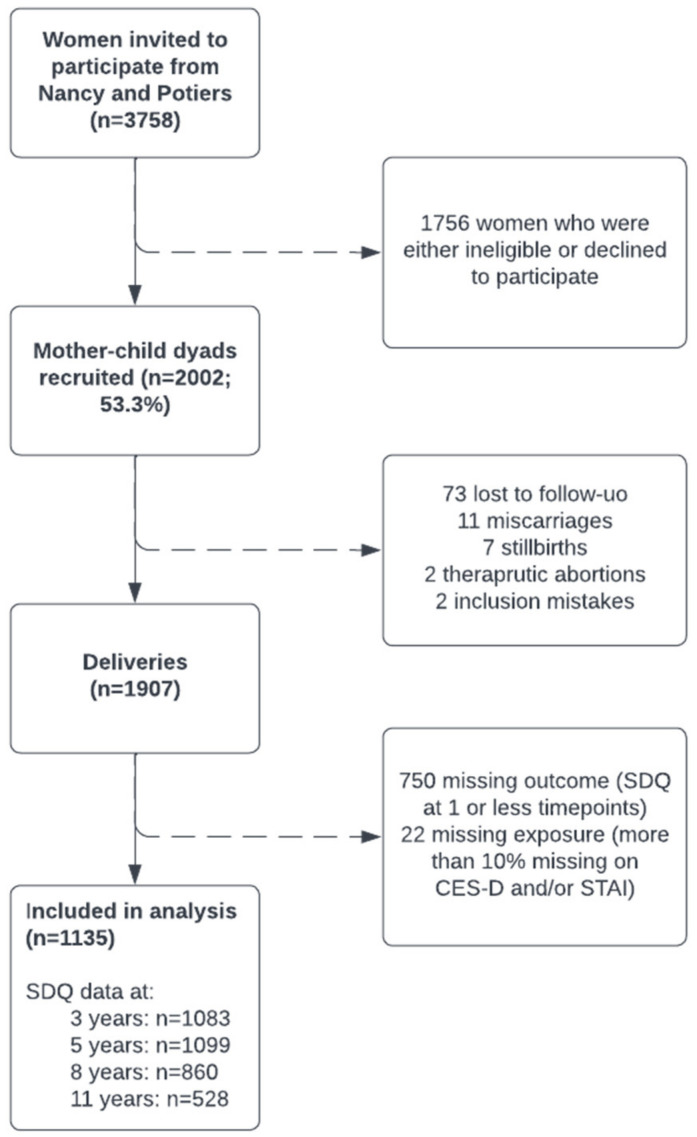
Flowchart of the study sample inclusion.

Analyses in this study were based on mother–child dyads where responses to at least 90% of questions on prenatal anxiety and depression symptomatology were available, as well as children’s behavioural scores in at least two of the four possible study waves at 3, 5.5, 8, and 11 years of age. Based on these criteria, 1135 mother–child dyads were included in the sample. A flowchart depicting the specifics of eligibility and participation of the sample is shown in Figure 1.

### 2.2. Variables

#### 2.2.1. Maternal Prenatal Depression and Anxiety

At the 24-week measurement, maternal symptoms of depression were assessed using the Centre for Epidemiological Studies Depression (CES-D) questionnaire (21), a 20-item questionnaire measuring the number of symptoms over the preceding week (range 0–60; Cronbach’s Alpha α = 0.852; 95% CI 0.828–0.872). A cut-off of 16 or above is generally recommended for inferring high levels of depressive symptomatology (22). Anxiety symptoms were measured at the same time using the State Anxiety Inventory (STAI) [31]. This 20-item questionnaire reflects how the individual feels at the moment they are completing the test (range from 20 to 80; Cronbach’s Alpha α = 0.91; 95% CI 0.897–0.92). Women scoring above the 80th percentile of the score distribution in the EDEN cohort were considered to have high levels of prenatal anxiety. The French versions of both the CES-D and STAI have good psychometric properties [32] and have been validated for use in pregnant populations. Given the high internal consistency values of the CES-D and STAI questionnaires in this sample, incomplete answers for those missing 10% or less of the questions were replaced with the person mean of all other answers. Based on their classification using the cut-off scores described above, women were identified as (1) neither anxious nor depressive symptomatology, (2) anxious symptomatology only, (3) depressive symptomatology only, or (4) both anxious and depressive symptomatology (comorbid group).

#### 2.2.2. Children’s Emotional and Behavioural Characteristics

Children’s emotional and behavioural patterns were assessed using the validated French version of the parent-reported Strengths and Difficulties Questionnaire (SDQ) [33]. The questionnaire was completed by the children’s mothers at ages 3, 5, 8, and 11. The SDQ is composed of 25 items comprising 5 scales: emotional symptoms, conduct problems, symptoms of hyperactivity/inattention, peer relationship problems, and prosocial behaviours. For each of the 5 scales, the score ranges from 0 to 10. The SDQ yields high internal consistency, test-retest stability, and parent-youth agreement of the various SDQ scales [34].

#### 2.2.3. Prenatal Mental Health Consultations

Both at inclusion and after birth, women self-reported whether they had consulted a psychologist or psychiatrist during pregnancy. Information was dichotomised as ‘reported a mental health consultation’ vs. ‘did not report a mental health consultation’.

#### 2.2.4. Covariates

In line with recommendations from Brookhart et al. [35] as well as Chesnaye et al. [36], all baseline covariates that could confound the relationship between the exposure and outcome were considered on the basis of the criteria for confounding [37].

Demographic measures included centre of recruitment, parity (nulliparous vs. multiparous), cohabiting with a partner (yes vs. no), family migration background (first generation, second generation, and native French), maternal age at birth (years), maternal and paternal education (years), maternal and paternal employment status (employed/studying vs. not), family income (above vs. below 1500 euros/month corresponding to the lowest quartile in the sample), and family financial difficulties (any difficulties with clothing, feeding or utilities, yes vs. no). Included psychosocial confounders were mother receiving support with practical problems from the partner (yes vs. no), support with practical problems from someone else in her network (yes vs. no), emotional support from the partner (yes vs. no), and emotional support from someone else in her network (yes vs. no). Maternal history of depression was considered through maternal reported antidepressant use prior to pregnancy (yes vs. no). Maternal experience of childhood adversities (any of the following: material deprivation, out-of-home placement, needing educational assistance, parental conflict, conflict with or between parents, violent home environment, or being a victim of physical violence) were dichotomised in one variable (yes vs. no). Finally, maternal and paternal childhood behavioural problems were considered via self-report (yes vs. no).

### 2.3. Statistical Methods

First, children’s available SDQ scores at the four data waves were used to classify their emotional and behavioural characteristics between the ages of 3 and 11 years into meaningful developmental trajectories, employing group-based trajectory modelling (GBTM) with a censored normal distribution. Multiple models were tested with different numbers of trajectories and combinations of polynomial shapes (intercept, linear, quadratic, and cubic). Bayesian information criterion (BIC) was used to guide model selection. In the choice of trajectories, the preference for an improvement of 2*∆BIC > 10 (Bayes factor) between consecutive models was paired with (a) a preference for a useful parsimonious model which fits the data well; (b) maximising the average posterior probability (App) value at >0.75 for each group; (c) adequate sample proportion in each group; (d) reasonably narrow confidence intervals; and (e) the odds of correct classification based on the posterior probabilities of group membership >5 for each group. In subsequent analyses, trajectory allocation for each of the SDQ subscales were used as dependent categorical variables.

Altogether, 15.4% of the participants were missing some data in the covariates. Thus, in line with recommendations by Bodner [38] for minimum number of imputations given to this proportion, 20 datasets were imputed using Fully Conditional Specification (FCS) implemented by the Multiple Imputation by Chained Equations (MICE) algorithm. Results were pooled from all MI datasets.

Second, multinomial logistic regressions were used to quantify the association between maternal prenatal anxiety and depression status with each of the SDQ subscale categories, taking the category with the least problematic characteristics (low trajectory for all four difficulties subscales and high trajectory for prosocial behaviours) as a reference group. Covariates associated with the outcome at *p* < 0.20 were used to calculate propensity scores to render exposure groups comparable on baseline characteristics. Propensity scores were incorporated into the analysis via inverse probability weighting (IPW) [39]. Following the application of the method, balance between exposed and unexposed groups was checked for all included baseline characteristics in each imputed dataset both before and after weighting via standardised mean differences (SMD) [40]. SMD below 0.1 can be considered negligible [36]. In cases where SMD remained above 0.1 in any of the twenty imputed datasets after the application of IPW, supplementary regressions were run to check for residual confounding by adding the variable to the pooled multivariate regression model. If there was a change in the beta coefficient of the exposure above 10% (indicating residual confounding), a doubly adjusted OR was presented in the final results [41]. If a covariate is rare, extreme weights can increase the variability of the treatment effect, leading to biased results. To address this, weights were curtailed at the 99th percentile [42].

Third, stratified analyses were conducted on whether the mother reported seeing a psychologist or psychiatrist during pregnancy. To avoid power problems due to few observations for some outcomes, women with any depressive or anxious symptoms were grouped together and compared to the group without any anxious or depressive symptoms.

Sensitivity analyses through standard multinomial regressions, including all relevant covariates on imputed datasets, were conducted by keeping the depression and anxiety scores continuous, as well as grouping all women with mental health symptoms against those with none.

Group-based trajectory modelling (GBTM) was executed via the Proc Traj package on SAS software (version 9.4; SAS Institute, Inc., Cary, NC, USA). All other analyses were conducted using R Studio (version 4.1.2).

## 3. Results

### 3.1. Description of the Study Population

The demographic characteristics of the sample are shown in Table 1, while the characteristics of the excluded participants from the cohort can be found in Supplementary Table S1. Compared to the excluded participants, the final sample was more likely to be primiparous, had lower rates of both maternal and paternal unemployment, and was less likely to report migrant background, low household income, and financial difficulties. The included women were less likely to report a lack of practical or emotional support, childhood adversity, maternal or paternal childhood behaviour problems, and antidepressant use prior to pregnancy. Included women were, on average older and had higher levels of education, both themselves and their partners as well. Of the 1135 women in the sample, 116 (10.2%) of women were classified as in the comorbid depressive and anxious symptoms group, 131 (11.5%) were classified as depressive only, and 83 (7.3%) were classified as anxious only. Overall, mothers classified as experiencing prenatal depression or anxiety were more likely to have less favourable profiles on key socioeconomic and social support characteristics such as fewer years of education, living in a household with income below EUR 1500 per month, and reporting financial difficulties. Classification with anxiety or depression was associated with a lower propensity to report the presence of practical support from partner, and emotional support from the partner and others in network, lower likelihood to live with the father of the child, having experienced adversity in childhood, and reporting behavioural problems in the childhoods of both parents. Women who experienced mental health symptoms during pregnancy were less likely to be primiparous but more likely to have a history of antidepressant use and to report visits to a psychologist or psychiatrist during pregnancy.

### 3.2. Child Emotional and Behavioural Trajectories

**Figure 2:**
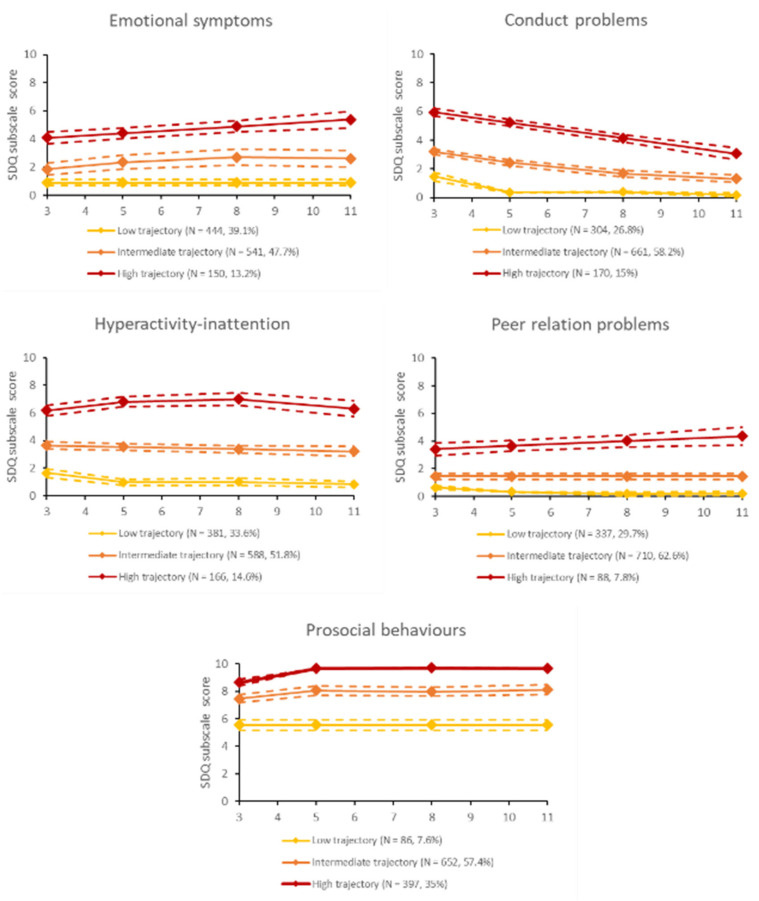
Trajectories of children’s emotional and behavioural symptom scores measured through parent-reported Strengths and Difficulty Questionnaires from ages 3 to 11 years (EDEN cohort study, *n* = 1135).

For all SDQ subscales, three-group trajectory models were chosen based on fit statistics, posterior probabilities, interpretability, and meaningful group sizes (see Supplementary Table S2A–E). Three-trajectory models for each of the subscales denote persistently low, intermediate, or high-level symptoms. Symptom trajectories between ages 3 and 11 are presented graphically along with 95% confidence intervals (CI) in Figure 2. Overall, 13.2% of the children were allocated to the trajectory indicating persistently high levels of emotional symptoms, 15% to the highest trajectory of conduct problems, 14.6% to high inattention-hyperactivity, and 7.8% to high levels of peer relationship problems group. A total of 7.6% followed a trajectory of persistently low levels of prosocial behaviours.

### 3.3. Prenatal Depression and Anxiety and Child Trajectories

In both the unadjusted or IPW-adjusted analyses (Table 2), children of mothers experiencing elevated levels of prenatal anxiety were at increased risk of following intermediate or high trajectories for the different outcomes.

In unadjusted analyses, children of mothers who experienced elevated levels of prenatal depression had an increased risk of following the high trajectory groups of conduct problems, inattention–hyperactivity, and peer relationship problems and a lower risk of following an intermediate prosocial behaviours trajectory. Following IPW adjustment for baseline characteristics, only the association with a lower likelihood to follow the intermediate trajectory of prosocial behaviours remained significant (OR_IPW_ = 0.64, 95% CI 0.42–0.97).

Prior to adjustment, children of mothers who reported comorbid anxiety and depressive symptoms were more likely to follow both the intermediate and high trajectories of emotional problems, as well as the high trajectories of conduct problems, inattention/hyperactivity, peer relationship problems, and the low trajectory of prosocial behaviours in comparison. Following control for key characteristics, children of mothers with comorbid anxiety and depression retained a higher probability of following high (OR_IPW_ = 2.46, 95% CI 1.26–4.80) and intermediate (OR_IPW_ = 1.85, 95% CI 1.09–3.16) trajectories of emotional problems and a high trajectory of conduct problems (OR_IPW_ = 2.02, 95% CI 1.01–4.17) in comparison to low symptom level trajectories.

A sensitivity analysis grouping all women with mental health symptoms against those with none found that the odds of allocation to high trajectories in all of the problem subscales and low trajectory of prosocial behaviours were higher in children of mothers who reported mental health symptoms in pregnancy (Supplementary Table S3). An additional sensitivity analysis using continuous depression and anxiety scores as exposures (Supplementary Table S4) revealed that increasing scores on either scale were associated with higher odds of following high trajectories of all emotional and behavioural symptoms.

### 3.4. Stratification by Prenatal Consultations with a Psychologist or Psychiatrist

Women who did not report any prenatal visits to a psychologist or psychiatrist were more likely to cohabit with the father of the child and were younger; they often further had a migrant background and reported the father of the child being unemployed/not studying. They were less likely to have a history of antidepressant use and childhood adversity than women who reported visits (Supplementary Table S5). Children’s SDQ subscale trajectory membership did not differ according to mothers reporting prenatal visits to a psychologist or psychiatrist or not (Supplementary Table S6). Overall, 16.8% of women with prenatal depressive symptoms, 13.7% of those with prenatal anxiety, and 22.4% with comorbid complaints indicated that they consulted a mental health specialist.

#### 3.4.1. Women Who Did Not Report Prenatal Consultations

For women who did not report any prenatal consultations with a psychologist or psychiatrist, the offspring of those with mental health symptoms were more likely to follow high trajectories of emotional symptoms (OR_IPW_ = 2.32, 95% CI 1.50–3.58), conduct problems (OR_IPW_ = 2.26, 95% CI 1.44–3.56), inattention/hyperactivity (OR_IPW_ = 2.06, 95% CI 1.33–3.20), and peer relationship problems (OR_IPW_ = 2.10, 95% CI1.21–3.63) compared to offspring of women without mental health symptoms (Table 3). They were additionally more likely to follow the low trajectory of prosocial behaviours (OR_IPW_ = 2.07, 95% CI 1.22–3.54).

#### 3.4.2. Women Who Reported Prenatal Consultations

Conversely, for children whose mothers reported consulting a psychologist or psychiatrist during pregnancy, exposure to prenatal depressive or anxious symptoms was associated with lower odds of following high trajectories of both externalising problems: conduct problems (OR_IPW_ = 0.09, 95% CI 0.02–0.48), and inattention–hyperactivity (OR_IPW_ = 0.15, 95% CI 0.03–0.77). It should be noted that only 19 mother–child dyads (19.4%) were allocated to the high conduct problems trajectory, while 11 (11.2%) were in the high inattention/hyperactivity trajectory. Associations were not significant for the internalising problems (emotional problems and peer relationship problems) or the prosocial scales of the SDQ (Table 3).

## 4. Discussion

The aim of this study was to investigate whether exposure to prenatal maternal depression or anxiety symptoms impacts longitudinal trajectories of child emotional and behavioural problems in a French sample from the general population. Additionally, we assessed whether a possible association differs for mothers who reported mental health support by visiting a psychologist or psychiatrist during pregnancy. We found that overall, children exposed to concurrently occurring prenatal maternal depression and anxiety were at higher risk of following longitudinal, adverse trajectories for emotional and conduct problems. When expectant mothers consulted with a mental health professional, their offspring had less chance to follow high trajectories for externalising, but not internalising, problems during childhood.

### 4.1. Child Trajectories of Emotional and Behavioural Problems

A 2001 WHO report indicated that 10–20% of all children present one or more mental or behavioural problems, which can be considered a public health issue with high morbidity that warrants specific policy and economic consideration [43]. However, most longitudinal studies on the evolution of child mental health problems over time have been limited by the late age of enrollment (mostly after school entry or even later in early adolescence), with few samples including children from early childhood onwards [28,44]. In our sample of children aged between 3 and 11, we identified for the different emotional and behavioural problems three trajectory groups (low-, intermediate-, or high-level symptoms) that were relatively stable over time. The proportion of children allocated to trajectories indicating higher levels of difficulties or lower levels of strengths (7.6–15%) can be considered clinically relevant [45], as well as in line with previously published studies in this sample [46,47,48]. Furthermore, it should be noted that conduct problems showed trajectories with decreasing scores over time as the children age, while the high trajectory of emotional problems shows an increasing trend. Consistent with previous research, these developmental cascades of internalising and externalising problems in childhood show that externalising problems are more prevalent in early childhood but diminish afterwards, while internalising problems become more prevalent from age 8 onwards [49,50,51].

### 4.2. Associations between Prenatal Mental Health and Child Outcomes

In the present sample, 21.7% of mothers reported prenatal depressive symptomatology, similar to the prevalence found by Gaugue-Finot, Devouche [52] in a French sample, but higher than commonly reported estimates for clinical depression (12–15%) [2,53]. The proportion of women classified as anxious (17.5%) is close to the prevalence of anxiety symptoms found in pregnant women in other studies [1,54,55]. In total, 116 women (10.2% of the total sample) were identified to have comorbid prenatal depressive and anxiety symptoms. This percentage is also higher than the 6.3% reported across various countries in a previous meta-analysis on co-occurring symptoms in the prenatal period [56].

As far as we know, our results are the first to model prenatal exposure to maternal mental health problems with longitudinal trajectories of emotional and behavioural development from preschool age to early adolescence. Our results did not show a significant association between anxiety-only symptoms and an increased risk of childhood emotional and behavioural problems, which is not in line with evidence from current meta-analyses [18,57]. Likewise, after adjustment for confounders, the impact of prenatal depressive symptoms on childhood emotional and behavioural problems also became non-significant. Thus, contrary to the existing literature, we showed that neither prenatal maternal depression nor anxiety symptoms separately are associated with an increased risk of childhood emotional and behavioural problems in our study. However, previous studies assessed associations between mothers experiencing prenatal mental health problems and later child outcomes measured at a specific point in development. Using trajectory modelling, we identified patterns of stability or change in child socioemotional and behavioural outcomes over time in relation to these exposures, which could account for some of the differences in results. Additionally, a sensitivity analysis looking at increasing depression and anxiety scores individually with each of the emotional and behavioural problems did indicate that both prenatal anxiety and depression were associated with the problem categories of child developmental trajectories, following an established pattern of stronger effect sizes with more severe depression and anxiety scores [57].

We expanded the existing literature by showing that, in particular, children exposed to comorbid maternal prenatal depressive and anxiety symptoms had higher odds of following high trajectories of emotional and conduct problems. Previously, Ibanez et al. [58] demonstrated with data from the same cohort that comorbid prenatal depressive and anxiety symptomatology led to worse neonatal outcomes than having either. While this needs to be confirmed by further studies, child emotional and behavioural characteristics over time could be associated with characteristics of the prenatal environment. The ‘Developmental Origins of Health and Disease (DOHaD) hypothesis’ [8] suggests that the in utero environment can have long-term consequences for infant and child development by setting probabilistic parameters for both adaptive and maladaptive outcomes. The body’s biological response to psychological stress is instigated by the sympathetic nervous system and the hypothalamic–pituitary–adrenal (HPA) axis, which alter the placental metabolism of maternal steroids [59]. The developing foetus is thus exposed to more stress biomarkers, such as glucocorticoids and pro-inflammatory cytokines [60], leading to increased activation of the foetal HPA axis and alterations in hippocampal neuronal development. Ultimately, these foetal changes could manifest in developmental outcomes such as greater cortisol and behavioural stress reactivity in the child [12]. Recent research shows indeed a potential mediating role of maternal biomarkers in the association between prenatal mental health problems and later child emotional and behavioural outcomes [61,62,63].

An alternative mechanism of transmission is the heritability of emotional and behavioural characteristics [64]. Although the study used both maternal and paternal self-reported childhood behavioural problems as a potential covariate, residual confounding cannot be ruled out. Finally, intergenerational stress transmission can occur via patterns of interpersonal interactions with a parent with mental health difficulties [9]. More than a third of women who experience prenatal depression go on to experience postnatal depression [65], which is highly implicated as an environmental factor affecting behavioural and emotional characteristics of the child [9,10,66]. Negative mental health symptoms during the completion of the self-report questionnaire may impact patterns of the way the parent reflects on both their own mental state and their child’s characteristics.

### 4.3. Prenatal Consultation of a Psychologist or Psychiatrist

Perinatal mood problems frequently go unnoticed in routine care [67]. Out of the 330 individuals with high symptom scores on depression and/or anxiety, only 58 (17.5%) reported visiting a psychologist or psychiatrist in this sample. Overall, 16.8% of women with prenatal depressive symptoms, 13.7% of those with prenatal anxiety, and 22.4% with comorbid complaints indicated that they consulted a mental health specialist. Of note is that our sample consisted of women from the general population in contrast to studies following clinical populations. Women with high levels of prenatal anxiety or depression who did not report seeking out a prenatal mental health consultation were, for example, less likely to have a history of antidepressant use, which could indicate lower levels of familiarity with the mental health care system. These findings seem to corroborate known facts about access to mental health services during the perinatal period, both in France and abroad [68,69]. Expectant mothers who experienced negative mood symptoms during pregnancy and actively sought out care may have found their symptoms to be a cause for concern. Searching for help could have indicated self-efficacy and awareness [70], allowing mothers to actively prioritise the development of their coping skills.

When left unaddressed through a prenatal psychologist or psychiatrist consultation, maternal negative mood symptoms during pregnancy were associated with children’s adverse developmental trajectories, while this increased risk was not present in the children of mothers who sought psychological support. While in our study, the numbers in the analyses were too low to draw reliable conclusions on the impact of consulting a mental health professional on the different classes of psychological difficulties, they do seem to indicate that this beneficial association might apply to all the studied child outcomes. Accessing mental health care seemed to buffer the potentially negative association between the mother’s mental condition and children’s emotional and behavioural difficulty trajectories. If left unaddressed, increased levels of anxiety and depressive symptomatology during pregnancy have previously been shown to stay highly stable throughout pregnancy and the postpartum [71] as well as being associated with adverse developmental outcomes, including lower receptive language and motor skills [11] and a higher prevalence of psychiatric problems, among children [66].

Thus, mental health consultations during pregnancy may have the potential to impact the developmental cascade. Detrimental effects of prenatal mental health problems may be mitigated by early interventions aimed at helping mothers classified as anxious or depressed deal with their symptoms. In the current sample, consultations during pregnancy could have made foetal exposure less acute and impactful, similar to evidence found in a meta-analysis by Sin and Lyubomirsky [72]. Mental health care visits during pregnancy might also have a postnatal spill-over effect by increasing mother–offspring attachment [73], dyadic affect regulation [74], and parenting [9], which appear to moderate the association between prenatal stress and offspring neurodevelopment. Thus, consulting a psychologist or psychiatrist during pregnancy could modify the effect itself; however, it may also be associated with mothers’ characteristics [75]. Higher levels of positive maternal mental health (self-esteem and self-efficacy) during pregnancy may buffer the associations between prenatal maternal stress and child internalising and externalising symptoms [76]. Self-efficacy is additionally associated with positive parenting practices [77], which promote child socioemotional functioning and may prevent the development of problematic emotional and behavioural characteristics. Further analyses are needed to characterise the full developmental cascade while maintaining awareness of its complexity and difficulties analysing it using rigid methods.

### 4.4. Strengths and Limitations

This study extends current knowledge by providing findings based on repeated measures and longitudinal assessments of children’s emotional and behavioural trajectories up to the age of eleven. Information was collected on a large number of factors, allowing for adjustment on numerous confounders, and rigorous methodology using propensity scores was applied through inverse probability weighting to render exposure groups strictly comparable [39] and account for selection and confounding factors. Information on both exposure and outcome was collected using validated scales.

Nevertheless, the findings should be interpreted in consideration of certain limitations. First, the study sample has, on average, higher educational attainment and higher income than the national average in France. Additionally, attrition occurred over the follow-up period, and the excluded participants differed from the included in nearly all socioeconomic and psychological characteristics measured. Selective attrition was addressed by applying statistical techniques such as IP weighting; however, it cannot be ruled out that associations may have been impacted by loss to follow-up. Measures of both exposure and outcome were derived from responses to self-report questionnaires, and as such, it is possible there may have been individual differences in reporting and recognising both mental states and child characteristics. Although future studies could benefit from simultaneous assessments derived from multiple reporters, parent-reported SDQ measures are generally considered valid and generally consistent with questionnaires filled out by external parties, such as teachers [78].

In the present study, the frequency and content of psychological care were not assessed, which prevents us from drawing conclusions about the reasons for which the women accessed care, as well as the type of care women actually received. Likewise, we did not have information on other sources of professional support that women might have accessed. Finally, despite some studies reporting consistency of symptoms of distress throughout pregnancy [10], information on this is lacking in our sample as the questionnaires were administered to the women at one specific time point during amenorrhea and thus represented a snapshot of momentary stress rather than persistent symptoms. However, given that mental states often stay overall stable throughout pregnancy, the results are presented on the assumption that, on average, the symptoms persist.

### 4.5. Recommendations for Research and Practice

In current practice, pregnant women with symptoms of mental health difficulties frequently go unnoticed in routine care [16], and as a result, few are able to access care and resources to manage their mental health difficulties. The results of this study suggest there could be significant benefits to incorporating comprehensive measures to optimise women’s psychological well-being into routine antenatal care. To facilitate conversations around sensitive issues, routine, standardised screening and clear referral pathways to mental health services need to be in place to address adverse mental health symptoms during pregnancy [67,79]. This type of care needs to be an active part of practice tailored to the family’s needs and characteristics. Indeed, there is substantial evidence in the literature on the potential effectiveness of tailored prenatal preventative programs that continue into the postnatal period, potentially targeting self-efficacy, maternal postnatal stress, mother–offspring attachment [73], and parenting behaviours [9].

Furthermore, providing this type of care from pregnancy onwards will indirectly impact child outcomes via preventive intervention directed at the mother [80]. France recently brought into law a compulsory prenatal appointment to create a space for expectant mothers to express any needs or difficulties. This consultation can be alone or with the co-parent and aims to navigate parents towards specialised services, such as mental health care services [81]. However, as underlined by Guedenay and Benarous [82], this consultation is not sufficient as its benefits are often undermined by the lack of continuity in care between the prenatal and postnatal periods. These authors suggest implementing a personalised follow-up book for each mother to remedy these issues, which would be an interesting path for future researchers to explore.

Future research can add to these findings in general by including both parents if mothers are in a relationship, as the literature has already shown how couples mutually influence each other [82,83]. To understand the mother’s prenatal mental health experience, it is important to explore how each parent within the couple adapts to the transition to parenthood and what support they provide each other during this time. Such a tailored and systemic approach is essential for the parents and their entourage to support the family through vulnerable times [67].

## 5. Conclusions

Children exposed to maternal prenatal comorbid depression and anxiety had a higher risk of following more problematic socioemotional and behavioural trajectories throughout childhood. This increased risk was reduced in the children of mothers who sought support through a prenatal psychologist or psychiatrist consultation. Considering the high prevalence of symptoms of prenatal mental health problems, the fact that they frequently go unnoticed in routine care, and their impact on offspring’s socioemotional and behavioural outcomes, this study provides further evidence that failure to address poor maternal mental health during pregnancy would be a missed opportunity for intervention and to ensure optimal outcomes and support for each individual across generations.

## Figures and Tables

**Table 1:** Characteristics of the study population by mental health status classification (*n* = 1135).

	Maternal Prenatal Mental Health				
Variables	No Symptoms (*n* = 805)	Anxious Symptoms Only (*n* = 83)	Depressive Symptoms Only (*n* = 131)	Comorbid (*n* = 116)	
	*n* (%)	*n* (%)	*n* (%)	*n* (%)	*p*-Value ^a^
Recruitment center (Nancy)	387 (48.07)	34 (40.96)	69 (52.67)	45 (38.79)	0.094
Primiparous (yes)	396 (49.19)	37 (44.50)	55 (41.98)	45 (38.79)	0.031 *
Mother unemployed and not studying	121 (15.03)	18 (21.69)	27 (20.61)	25 (21.55)	0.231
Father unemployed and not studying	34 (4.22)	3 (3.61)	4 (3.05)	7 (6.03)	0.074
Migrant background					0.199
None	706 (87.70)	74 (89.16)	111 (84.73)	94 (81.03)	
Second generation	65 (8.07)	5 (6.02)	16 (12.21)	15 (12.93)	
First generation	16 (1.99)	1 (1.20)	3 (2.29)	5 (4.31)	
Household income <1500 EUR/month	70 (8.70)	13 (15.66)	14 (10.69)	22 (18.97)	0.018 *
At least one financial difficulty	29 (3.60)	7 (8.43)	12 (2.76)	17 (14.66)	<0.001 ***
Antidepressant use before pregnancy	25 (3.11)	4 (4.82)	7 (5.34)	20 (17.24)	<0.001 ***
Not receiving practical support (partner)	57 (7.08)	9 (10.84)	14 (10.69)	11 (9.48)	0.010 **
Not receiving practical support (other)	122 (15.16)	18 (21.69)	30 (22.90)	18 (15.52)	0.085
Not receiving emotional support (partner)	13 (1.61)	4 (4.82)	5 (3.82)	7 (6.03)	0.002 **
Not receiving emotional support (other)	36 (4.47)	7 (8.43)	11 (8.40)	12 (10.34)	0.027 *
Living with father of the child (no)	15 (1.86)	4 (4.82)	10 (7.63)	8 (6.90)	0.001 ***
Childhood adversity (yes)	176 (21.86)	19 (22.89)	44 (33.59)	46 (39.66)	<0.001 ***
Childhood behaviour problems (mother)	35 (4.35)	3 (3.61)	12 (9.16)	15 (12.93)	0.009 **
Childhood behaviour problems (father)	72 (8.94)	6 (7.23)	21 (16.03)	6 (5.17)	0.028 *
Child sex (female)	363 (45.09)	37 (44.58)	73 (55.73)	59 (50.86)	0.109
Consulted psychologist/psychiatrist in pregnancy	40 (4.97)	14 (16.87)	18 (13.74)	26 (22.41)	<0.001 ***
	**Mean (SD)**	**Mean (SD)**	**Mean (SD)**	**Mean (SD)**	***p*-value ^b^**
Maternal age (years)	30.52 (4.60)	30.96 (4.58)	30.29 (4.88)	31.69 (5.17)	0.067
	**Median (IQR)**	**Median (IQR)**	**Median (IQR)**	**Median (IQR)**	***p*-value ^c^**
Maternal education (years)	14 (12–17)	14 (11–17)	14 (12–17)	12 (11–14)	<0.001 ***
Paternal education (years)	14 (11–17)	12 (11–17)	12 (11–17)	12 (11–14)	0.410

^a^ Fisher’s exact test; ^b^ One-way ANOVA; ^c^ Kruskal–Wallis one-way analysis of variance. * *p* < 0.05; ** *p* < 0.01; *** *p* < 0.001. Cell counts may vary due to missing observations.

**Table 2:** Unadjusted and IPW-adjusted multinomial regressions comparing associations with child’s trajectories in each group with any mental health problem symptoms (depressive only, anxious only, comorbid depressive and anxious) to associations in the reference group (no symptoms (*n* = 805)).

		Anxious, Non-Depressive (*n* = 83)				Depressive, Non-Anxious (*n* = 131)				Depressive and Anxious (*n* = 116)			
SDQ Subscales		Unadjusted		Adjusted		Unadjusted		Adjusted		Unadjusted		Adjusted	
		OR	95% CI	OR	95% CI	OR	95% CI	OR	95% CI	OR	95% CI	OR	95% CI
Emotional symptoms	Low	Ref		Ref		Ref		Ref		Ref		Ref	
	Intermediate	1.12	(0.68–1.84)	1.01	(0.59–1.71)	1.29	(0.86–1.93)	1.21	(0.78–1.87)	**1.67**	**(1.06–2.63)**	**1.85**	**(1.09–3.16)**
	High	1.53	(0.77–3.05)	1.50	(0.73–3.08)	1.70	(0.97–3.00)	1.63	(0.90–2.96)	**2.74**	**(1.54–4.88)**	**2.46**	**(1.26–4.80)**
Conduct problems	Low												
	Intermediate	1.24	(0.72–2.16)	1.04	(0.58–1.84)	1.14	(0.72–1.80)	0.98	(0.60–1.61)	1.23	(0.76–2.01)	1.10	(0.63–1.93)
	High	1.85	(0.90–3.80)	1.40	(0.65–3.01)	**2.35**	**(1.34–4.10)**	1.65	(0.90–3.02)	**2.53**	**(1.40–4.59)**	**2.02**	**(1.01–4.07)**
Inattention-hyperactivity	Low												
	Intermediate	1.33	(0.79–2.23)	1.22	(0.71–2.10)	1.48	(0.96–2.29)	1.49	(0.93–2.38)	1.35	(0.86–2.12)	1.30	(0.76–2.22)
	High	1.65	(0.82–3.31)	1.54	(0.72–3.29)	**2.16**	**(1.24–3.77)**	1.63	(0.89–2.98)	**2.03**	**(1.14–3.63)**	1.83	(0.86–3.86)
Peer relation problems	Low												
	Intermediate	1.09	(0.65–1.81)	1.07	(0.62–1.86)	1.50	(0.97–2.33)	1.44	(0.90–2.29)	1.33	(0.84–2.12)	1.02	(0.59–1.74)
	High	1.63	(0.70–3.84)	1.42	(0.55–3.63)	**2.12**	**(1.04–4.35)**	1.63	(0.76–3.47)	**2.63**	**(1.31–5.26)**	1.85	(0.84–4.08)
Prosocial behaviours	Low	1.98	(0.88–4.47)	1.96	(0.80–4.78)	1.43	(0.74–2.76)	1.32	(0.66–2.65)	**2.56**	**(1.25–5.27)**	2.24	(0.98–5.13)
	Intermediate	1.13	(0.68–1.85)	1.07	(0.63–1.80)	**0.66**	**(0.45–0.98)**	**0.64**	**(0.42–0.97)**	1.56	(0.99–2.45)	1.32	(0.80–2.19)
	High												

Bivariate and IPW-adjusted multinomial regressions (95% CI). IPW, Inverse Probability Weight; SDQ, Strengths and Difficulties Questionnaire; Ref, reference group. Significant associations in bold.

**Table 3:** Stratified analysis by consultations with a psychologist or psychiatrist during pregnancy or not). IPW-adjusted multinomial regressions comparing associations between exposure to any prenatal negative mood symptoms and odds of following intermediate and high trajectories of emotional and behavioural problems in both groups. In both analyses, the children with no exposure to negative mental health symptoms are taken as a reference group.

SDQ Subscales		Did Not Report a Consultation (*n* = 1037; Any Negative Mental Health Symptoms in 272 (26.2%))	Reported Mental Health Consultations during Pregnancy (*n* = 98; Any Negative Mental Health Symptoms in 58 (59.2%))
		OR (95% CI)	OR (95% CI)
Emotional symptoms	Low	Ref	Ref
	Intermediate	1.27 (0.92–1.76)	1.12 (0.41–3.03)
	High	**2.32 (1.50–3.58)**	0.38 (0.08–1.80)
Conduct problems	Low	Ref	Ref
	Intermediate	1.16 (0.81–1.65)	0.37 (0.11–1.22)
	High	**2.26 (1.44–3.56)**	**0.09 (0.02–0.48)**
Inattention- hyperactivity	Low	Ref	Ref
	Intermediate	**1.49 (1.06–2.10)**	**0.32 (0.12–0.84)**
	High	**2.06 (1.33–3.20)**	**0.15 (0.03–0.77)**
Peer relation problems	Low	Ref	Ref
	Intermediate	1.36 (0.96–1.93)	0.93 (0.35–2.48)
	High	**2.10 (1.21–3.63)**	1.79 (0.18–17.54)
Prosocial behaviours	Low	**2.07 (1.22–3.54)**	0.47 (0.17–1.46)
	Intermediate	1.06 (0.77–1.45)	0.47 (0.18–1.20)
	High	Ref	Ref

IPW-adjusted multinomial regressions. SDQ, Strengths and Difficulties Questionnaire; Ref, reference; IPW, Inverse Probability Weight. Odds Ratios that are significant at CI 95% are in bold.

## Data Availability

The data analyzed during the current study are not publicly available due to ethical and legal restrictions. This is because the present study includes an important number of variables that, together, could be used to identify the participants based on a few key characteristics and then be used to have access to other personal data. Therefore, the French ethical authority strictly forbids making such data freely available. However, they can be obtained on reasonable request from the EDEN scientific board.

## Supplementary Materials

The following supporting information can be downloaded at: https://www.mdpi.com/article/10.3390/jcm12031120/s1, Table S1: Characteristics of the EDEN cohort by whether they were included in the present study; Table S2: Comparison of model parameters for one-, two-, three-, four- and five-group trajectory models. Bayesian Information Criterions (BIC) and average posterior probabilities (App); Table S3: Any maternal prenatal mental health symptoms and children’s emotional and behavioural trajectories from ages 3 to 11 (*n* = 1135); Table S4: Continuous prenatal depression and anxiety scores and children’s emotional and behavioural trajectories from ages 3 to 11 (*n* = 1135); Table S5: Characteristics of the EDEN cohort by whether they consulted a psychologist or psychiatrist during pregnancy; Table S6: SDQ subscale trajectory membership by consultations with a psychiatrist during pregnancy.
