# Supplementary material for: Maternal Mental Health Care Matters: The Impact of Prenatal Depressive and Anxious Symptoms on Child Emotional and Behavioural Trajectories in the French EDEN Cohort
Source: J Clin Med. 2023 Jan 31;12(3):1120. doi: 10.3390/jcm12031120 (PMC9917852; doi:10.3390/jcm12031120)
Supplement: Supplementary file 1 [file jcm-12-01120-s001.zip › jcm-2139942-supplementary.pdf]

Supplemental Table S1. Characteristics of the EDEN cohort by whether they were included in the present study.

	Included (n=1135)		Excluded (n=867)		
	**n**	**%**	**n**	**%**	***P-value*[a]**
Centre of recruitment (Nancy)	535	47.14	498	57.44	*<0.001****
Primiparous (yes)	533	47.04	315	40.91	*0.009***
Mother unemployed and not studying	191	16.95	235	30.09	*<0.001****
Father unemployed and not studying	48	4.31	76	10.13	*<0.001****
Migrant background					*<0.001****
None	985	88.66	629	81.06	
Second generation	101	9.09	95	12.24	
First generation	25	2.25	52	6.70	
Household income <1500 €/month	119	10.48	208	26.53	*<0.001****
At least one financial difficulty (clothing, feeding, utilities)	65	5.77	105	13.44	*<0.001****
Antidepressant use before pregnancy	56	4.97	67	8.61	*0.002***
Practical support (partner)	91	8.10	102	13.14	*<0.001****
Practical support (someone else)	188	16.64	137	17.47	*0.66*
Emotional support (partner)	29	2.59	63	8.12	*<0.001****
Emotional support (someone else)	66	5.85	94	11.99	*<0.001****
Not living with father of the child	37	3.28	85	10.84	*<0.001****
Childhood adversity (mother)	285	25.51	283	36.61	*<0.001****
Childhood behaviour problems (mother)	65	5.79	77	9.85	*0.001****
Childhood behaviour problems (father)	105	9.95	99	14.18	*0.008***
Child sex (Female)	532	46.87	371	42.79	*0.543*
Known visits to a psychiatrist during pregnancy	98	8.63	83	9.57	*0.480*
	**Mean**	**SD**	**Mean**	**SD**	**P-value[b]**
Maternal age (years)	30.64	4.70	29.42	4.91	*<0.001****
	**Median**	**IQR**	**Median**	**IQR**	**P-value[c]**
Maternal education (years)	14	12.0 – 17.0	12	11.0-14.0	*<0.001****
Paternal education (years)	14	11.0 – 17.0	12	11.0-14.0	*<0.001****

[a] Fisher's exact test; [b] Independent samples t-test; [c] Unpaired Two-Samples Wilcoxon Test
* p<0.05; ** p<0.01; *** p<0.001

Supplemental Table S2(A-E). Comparison of model parameters for one-, two-, three-, four- and five-group trajectory models. Bayesian Information Criterions (BIC) and average posterior probabilities (App).

(A) Trajectories of emotional symptoms

**Model**	**BIC**	App group 1	App group 2	App group 3	App group 4	App group 5
1-group	-7054.05	1				
2-group	-6791.36	0.918	0.874			
3-group	-6752.94	0.807	0.764	0.810		
4-group	-6747.23	0.804	0.734	0.661	0.733	
5-group	-6739.71	0.688	0.775	0.662	0.638	0.792

(B) Trajectories of inattention-hyperactivity

**Model**	**BIC**	App group 1	App group 2	App group 3	App group 4	App group 5
1-group	-7946.89	1				
2-group	-7523.02	0.928	0.896			
3-group	-7383.14	0.884	0.861	0.883		
4-group	-7260.47	0.781	0.794	0.796	0.865	
5-group	-7372.88	0.779	0.773	0.754	0.717	0.795

(C) Trajectories of peer relation problems

**Model**	**BIC**	App group 1	App group 2	App group 3	App group 4	App group 5
1-group	-6058.31	1				
2-group	-5883.52	0.892	0.825			
3-group	-5825.60	0.785	0.846	0.831		
4-group	-5815.20	0.753	0.598	0.846	0.830	
5-group	-5808.39	0.621	0.648	0.770	0.813	0.781

(D) Trajectories of conduct problems

**Model**	**BIC**	App group 1	App group 2	App group 3	App group 4	App group 5
1-group	-7017.98	1				
2-group	-6707.20	0.910	0.864			
3-group	-6579.45	0.846	0.890	0.890		
4-group	-6650.91	0.801	0.631	0.852	0.846	
5-group	-6640.51	0.831	0.784	0.605	0.768	0.805

(E) Trajectories of prosocial behaviours

**Model**	**BIC**	App group 1	App group 2	App group 3	App group 4	App group 5
1-group	-6615.77	1				
2-group	-6344.70	0.874	0.886			
3-group	-6277.06	0.822	0.853	0.851		
4-group	-6297.90	0.812	0.738	0.761	0.773	
5-group	-6299.46	0.900	0.776	0.754	0.758	0.580

Supplemental Table S3. Any maternal prenatal mental health symptoms and children's emotional and behavioural trajectories from ages 3 to 11 (n=1135)

		Anxious or depressed (n=330) vs not (n=805)	
**SDQ subscales**		**Unadjusted OR 95% CI**	**IPW OR 95% CI**
Emotional symptoms	Low	Ref	Ref
	Intermediate	**1.35 (1.02-1.80)**	1.30 (0.97-1.75)
	High	**1.96 (1.32-2.91)**	**1.94 (1.28-2.92)**
Conduct problems	Low	Ref	Ref
	Intermediate	1.20 (0.88-1.64)	1.02 (0.74-1.42)
	High	**2.28 (1.53-3.41)**	**1.67 (1.09-2.56)**
Inattention-Hyperactivity	Low	Ref	Ref
	Intermediate	**1.39 (1.04-1.87)**	1.23 (0.89-1.68)
	High	**1.98 (1.34-2.93)**	**1.58 (1.03-2.43)**
Peer relation problems	Low	Ref	Ref
	Intermediate	1.32 (0.98-1.78)	1.16 (0.85-1.56)
	High	**2.15 (1.32-3.52)**	**1.75 (1.04-2.95)**
Prosocial behaviours	Low	**1.86 (1.15-3.00)**	1.61 (0.97-2.66)
	Intermediate	1.01 (0.76-1.33)	0.91 (0.68-1.22)
	High	Ref	Ref

Bivariate and IPW- adjusted multinomial regressions (95% CI).

SDQ, Strengths and Difficulties Questionnaire; Ref, reference group; OR Odds Ratio

Significant odds Ratios in bold.

Supplemental Table S4. Continuous prenatal depression and anxiety scores and children's emotional and behavioural trajectories from ages 3 to 11 (n=1135)

		Depressive scores (CESD)				Anxiety scores (STAI)			
**SDQ subscales**		Unadjusted analysis		Covariate-adjusted		Unadjusted analysis		Covariate-adjusted	
		**r**	**p**	**r**	**p**	**r**	**p**	**r**	**p**
Emotional symptoms	Low	Ref		Ref		Ref		Ref	
	Intermediate	0.031	*0.001****	0.026	*0.008***	0.040	*0.001****	0.021	*0.006***
	High	0.063	*<0.001****	0.061	*<0.001****	0.024	*<0.001****	0.038	*<0.001****
Conduct problems	Low	Ref		Ref		Ref		Ref	
	Intermediate	0.032	*0.003***	0.023	*0.030**	0.016	*0.054*	0.010	*0.211*
	High	0.057	*<0.001****	0.041	*0.003***	0.039	*<0.001****	0.030	*0.005***
Inattention-Hyperactivity	Low	Ref		Ref		Ref		Ref	
	Intermediate	0.028	*0.004***	0.019	*0.057*	0.012	*0.109*	0.013	*0.102*
	High	0.054	*<0.001****	0.042	*0.002***	0.041	*<0.001****	0.030	*0.005***
Peer relation problems	Low	Ref		Ref		Ref		Ref	
	Intermediate	0.025	*0.010***	0.010	*0.236*	0.012	*0.109*	0.003	*0.669*
	High	0.051	*0.001****	0.036	*0.035**	0.041	*<0.001****	0.032	*0.012**
Prosocial behaviours	Low	0.039	*0.009***	0.027	*0.085*	0.041	*<0.001****	0.033	*0.007***
	Intermediate	0.007	*0.434*	0.002	*0.804*	0.018	*0.016**	0.016	*0.040*
	High	Ref		Ref		Ref		Ref	

Bivariate and adjusted multinomial regressions (95% CI). SDQ, Strengths and Difficulties Questionnaire; Ref, reference.

* p<0.05; ** p<0.01; *** p<0.001

Supplemental Table S5. Characteristics of the EDEN cohort by whether they consulted a psychologist or psychiatrist during pregnancy

	Reported mental health consultations (n=98)		Did not report a consultation (n=1037)		
	**n**	**%**	**n**	**%**	***P-value*** [a]
Centre of recruitment (Nancy)	50	51.02	485	46.77	*0.459*
Primiparous (yes)	44	45.36	489	47.20	*0.186*
Mother unemployed and not studying	12	12.24	179	17.40	*0.391*
Father unemployed and not studying	8	8.51	40	33.33	*0.023**
Migrant background					*0.029**
None	78	81.25	907	89.34	
Second generation	17	17.71	84	8.28	
First generation	1	1.04	24	2.36	
Household income <1500 €/month	10	10.20	109	10.57	*1*
At least one financial difficulty	5	5.15	60	6.19	*0.757*
Antidepressant use before pregnancy	24	24.49	32	3.11	*<0.001****
Practical support (partner)	7	8.14	84	8.17	*0.427*
Practical support (someone else)	21	21.65	167	16.17	*0.163*
Emotional support (partner)	3	3.13	26	2.54	*0.485*
Emotional support (someone else)	7	7.22	59	5.72	*0.352*
Not living with father of the child	7	7.22	30	2.91	*0.047**
Childhood adversity (mother)	36	37.11	249	24.41	*0.023**
Childhood behaviour problems (mother)	11	11.22	54	5.27	*0.051*
Childhood behaviour problems (father)	12	13.19	93	9.65	*0.498*
	**Mean**	**SD**	**Mean**	**SD**	**P-value** [b]
Maternal age (years)	32.05	4.99	30.51	4.66	*0.004***
	**Median**	**IQR**	**Median**	**IQR**	**P-value** [c]
Maternal education (years)	14.0	12.0-17.0	14.0	12.0-17.0	*0.392*
Paternal education (years)	14.0	12.0-17.0	12.0	11.0-17.0	*0.128*

[a] Fisher's exact test; [b] Independent samples t-test; [c] Unpaired Two-Samples Wilcoxon Test
* p<0.05 ; ** p<0.01; *** p<0.001
Cell counts may vary due to missing observations

Supplemental Table S6. SDQ subscale trajectory membership by consultations with a psychologist or psychiatrist during pregnancy

	Reported mental health consultations (n=98)		Did not report a consultation (n=1037)		
	**n**	**%**	**n**	**%**	**P-values[a]**
Emotional symptoms					*0.884*
Low trajectory (N = 444, 39.1%)	36	36.73	408	39.34	
Intermediate trajectory (N = 541, 47.7%)	49	50.00	492	47.44	
High trajectory (N = 150, 13.2%)	13	13.27	137	13.21	
Conduct problems					*0.336*
Low trajectory (N = 304, 26.8%)	22	22.45	282	27.19	
Intermediate trajectory (N = 661, 58.2%)	57	58.16	604	58.24	
High trajectory (N = 170, 15%)	19	19.39	151	14.56	
Inattention - hyperactivity					*0.628*
Low trajectory (N = 381, 33.6%)	34	34.69	347	33.46	
Intermediate trajectory (N = 588, 51.8%)	53	54.08	535	51.59	
High trajectory (N = 166, 14.6%)	11	11.22	155	14.95	
Peer relationship problems					*0.310*
Low trajectory (29.7%)	35	35.71	302	29.12	
Intermediate trajectory (62.6%)	58	59.18	652	62.87	
High trajectory (7.8%)	5	5.10	83	8.00	
Prosocial behaviours					*0.316*
Low trajectory (N = 86, 7.6%)	7	7.14	79	7.62	
Intermediate trajectory (N = 652, 57.4%)	50	51.02	602	58.05	
High trajectory (N = 397, 35%)	41	41.84	356	34.33	

[a] Fisher's exact test, * p<0.05 ; ** p<0.01; p<0.001***
